# Programmatic Evaluation of a Combined Antigen and Antibody Test for Rapid HIV Diagnosis in a Community and Sexual Health Clinic Screening Programme

**DOI:** 10.1371/journal.pone.0028019

**Published:** 2011-11-22

**Authors:** Miriam Taegtmeyer, Peter MacPherson, Kathy Jones, Mark Hopkins, Jay Moorcroft, David G. Lalloo, Anu Chawla

**Affiliations:** 1 Liverpool School of Tropical Medicine, Liverpool, United Kingdom; 2 Wellcome Trust Tropical Centre, University of Liverpool, Liverpool, United Kingdom; 3 The Liverpool Centre for Sexual Health, Royal Liverpool and Broadgreen University Hospitals NHS Trust, Liverpool, United Kingdom; 4 Liverpool Specialist Virology Centre, Royal Liverpool and Broadgreen University Hospitals NHS Trust, Liverpool, United Kingdom; Duke-NUS Graduate Medical School, Singapore

## Abstract

**Background:**

A substantial proportion of HIV-infected individuals in the UK are unaware of their status and late presentations continue, especially in low prevalence areas. Fourth generation antigen/antibody rapid test kits could facilitate earlier diagnosis of HIV in non-clinical settings but lack data on performance under programmatic conditions.

**Methods and Findings:**

We evaluated the performance of Determine HIV-1/2 Ag/Ab Combo Test (Determine Combo), a rapid test with indicators for both HIV antibodies and p24 antigen, in participants recruited from community outreach and hospital-based sexual health clinics. HIV infection was confirmed using laboratory enzyme-linked immunosorbent assay (EIA), Line Immuno Assay (LIA) and quantitative polymerase chain reaction (PCR). In total, 953 people underwent HIV testing. HIV antibody (Ab) prevalence was 1.8% (17/953). Four false positive rapid tests were identified: two antibody and two p24 antigen (Ag) reactions. Of participants diagnosed as HIV Ab positive, 2/17 (12%) were recent seroconverters based on clinical history and HIV antibody avidity test results. However, none of these were detected by the p24 antigen component of the rapid test kit. There were no other true positive p24 Ag tests.

**Conclusion:**

These data lend support to an increasing body of evidence suggesting that 4th generation rapid HIV tests have little additional benefit over 3rd generation HIV kits for routine screening in low prevalence settings and have high rates of false positives. In order to optimally combine community-based case-finding among hard-to-reach groups with reliable and early diagnosis 3rd generation kits should be primarily used with laboratory testing of individuals thought to be at risk of acute HIV infection. A more reliable point of care diagnostic is required for the accurate detection of acute HIV infection under programmatic conditions.

## Introduction

Fourth generation HIV antibody-antigen combination tests are increasingly used in UK laboratory settings to ensure reliable laboratory diagnosis of acute HIV infection, since the antibody may not be detected during the so-called “seroconversion window period” [1], [2]. Simple rapid point of care HIV antibody tests are well established in voluntary counselling and testing (VCT) centres globally [3] and increasingly used in community settings in UK [4], [5]. However, there is little field data on the additional benefit of p24 antigen rapid tests in community programmes. With the global scale up of the use of rapid tests, reliable point of care detection of acute HIV infection in non-laboratory settings may have additional public health benefits in identifying acute infection earlier [6].

Kit manufacturers have developed a 4th generation point of care antigen/antibody kit (Determine HIV 1/2 Ag/Ab Combo; Alere). This has performed well in published data against known panels of seroconverters [7] with the p24 antigen detection preceding the development of antibody, by an average of 5 to 9 day [8]. However, it has performed less well in field evaluations in London [9] and Malawi [10] with both reporting a low sensitivity for the p24 component.

Case-finding of individuals with recent HIV infection is an important clinical and public health intervention [6]. Acute HIV infection is under-recognised with, in one UK study, approximately half of patients experiencing non-specific symptoms of HIV seroconversion and attending primary care or emergency departments failing to be diagnosed [11]. The inclusion of p24 antigen on rapid HIV test kits leads to a shortening of the diagnostic window period and the ease of use of these kits could lead to a greater detection of acute infection in clinical and community sites where a seroconversion illness may have otherwise been missed.

Liverpool has a low prevalence of HIV infection [12] but continues to have a significant number of late presentations, with an estimated one-third of HIV positive individuals undetected in the community [13]. Presentations of acute HIV infection to clinicians often go unrecognised [14]. Neither the hospital-based sexual health clinic nor the existing outreach and community programmes were offering point of care HIV testing before this study. We set out to evaluate the utility of fourth generation HIV tests under programmatic conditions in such settings and to make recommendations for policy makers.

## Methods

We offered rapid HIV testing with Determine HIV 1/2 Ag/Ab Combo to individuals seeking care at a variety of existing community services for intravenous drug users (IVDUs), men who have sex with men (MSM), asylum seekers and sex workers, as well as communities of UK Africans through church groups. In addition, The Liverpool Centre for Sexual Health, a hospital genitourinary medicine (GUM) clinic, conducted testing for outpatient clinic attendees.

Our target group for HIV testing included individuals who felt they may have been at- risk of HIV. However, we recognised that all participants may not have had similar levels of HIV risk behaviour. Therefore, participants who self-identified themselves as any of the following groups were classified as being “high-risk”: MSM; current or previous history of IVDU; originating from a high prevalence country; having bought or sold sex; reported being raped; or having an HIV-positive partner. Presumed acute HIV infection was defined as a participant who had laboratory confirmed HIV infection; and had a documented negative laboratory HIV test at our centre within three months prior to undergoing rapid HIV testing in the study; and had a clinical history consistent with risk exposure in the previous three months.

Based on the World Health Organization's recommendations for rapid HIV testing programmes, a serial testing algorithm was used [15]. Testing was conducted according to manufacturer's instructions by trained nurses and health care assistants already employed in community outreach or GUM settings. Training and certification of competency was done by the virology laboratory against a panel of known sera. The laboratory also provided samples to the sites for regular internal and external quality assurance including a 6-monthly blinded proficiency panel. Test results were recorded on a standardised register in all sites and visually verified by a second reader before being discarded. Tests were deemed “invalid” if the control line failed to appear by the end of the read time as stipulated in the manufacturer's instructions.

Invalid rapid tests that failed to resolve on immediate retesting and all reactive rapid tests had a venous sample sent to the laboratory for confirmatory testing. Antibody reactive tests or invalid rapid tests were retested using a confirmatory fourth- generation EIA followed by another confirmatory test Line Immuno assay (LIA, Inno-Lia HIV-1/HIV-2 Ab Innogenetics, Belgium). P24 antigen reactive rapid tests were additionally retested in the laboratory using two separate RNA viral load Cobas TaqMan HIV-1 Test, (Roche Diagnostics, UK) measurements a week apart. Detailed risk histories and any previous stored samples from the same individuals (when available) were reviewed for all participants with reactive or invalid results on point of care testing. Samples from individuals meeting our clinical criteria for seroconversion were also tested by HIV antibody avidity assay subject to availability of sample with a low avidity index being considered consistent with recent infection [16].

Ethical approval was obtained from the institutional review boards of Liverpool School of Tropical Medicine, NHS Sefton and Cheshire Research Ethics Committee Liverpool and the Royal Liverpool University and Broadgreen Hospitals Trust. Informed verbal consent for HIV testing was obtained from all participants and recorded in participant's clinical notes at the GUM clinic and in study logbooks at community-based sites. Written consent was not required by the research ethic committees as this study undertook post-marketing assessment of test kit performance. The institutional review boards approved the verbal consent procedures for HIV testing. All participants diagnosed HIV positive were referred for comprehensive HIV care (including assessment for antiretroviral therapy) at the genitourinary medicine clinic at the Royal Liverpool and Broadgreen Hospital.

## Results

Data from 953 individuals tested in nine months, from September 2009 to July 2010, were included in this analysis. More men (659/946, 69.7%) than women (287/946, 30.3%) were tested and the median age was 29 years (interquartile range (IQR): 23–38 years). Approximately 70% (435/641) were white British. Complete self-reported risk data were available on 927/953 (97.3%). In total, 517/927 (55.7%) had one or more behaviours identified that may have put them at “high-risk” of HIV (Table 1): 133/926 (14.4%) originated from a high prevalence country; 264/931 (28.4%) self- identified as men who have sex with men; intravenous drug use was identified as a risk in 82/927 (8.6%); 38/927 (4.1%) had bought or sold sex; 28/927 (3.0%) people requested testing because they had an HIV positive partner; and 18/927 (1.9%) after being raped.

**Table 1:** Baseline characteristics and reported risk exposure of participants.

Characteristic	N (%)§
Male	659/946 (69.7)
Median Age (IQR)	29 (23–38)
Any identified high risk factor*	517/927 (55.8%)
*Risk summary categories (Not mutually exclusive: one case can be represented in multiple categories)*	
UK African	133/927 (14.3%)
Intravenous drug user	82/927 (8.8%)
MSM	264/927 (28.5%)
CSW	38/927 (4.1%)
HIV-positive partner	28/927 (3.0%)
Reported rape	18/927 (1.9%)

*43 participants had more than one identified risk factors.

§ Denominator varies due to missing data in some categories.

MSM: men-who-have-sex-with-men CSW: commercial sex work (bought or sold sex).

**Figure 1:**
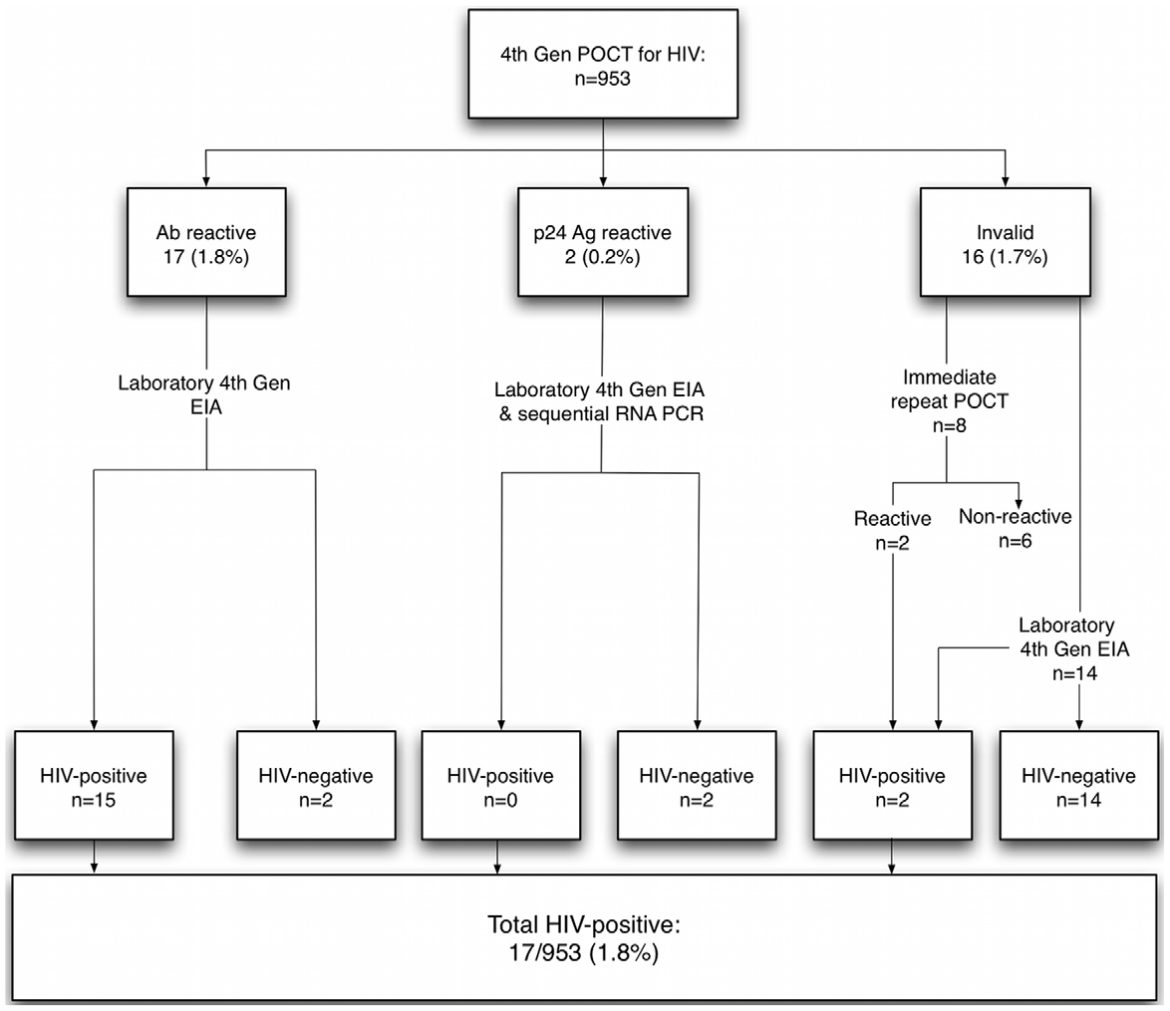
Fourth generation HIV rapid test and laboratory results. POCT: Point of care rapid test for HIV, EIA: Enzyme-linked immunosorbent assay, Ab: antibody, Ag: antigen, Gen: generation.

Seventeen new cases of HIV were detected, 16 among “high-risk” individuals and one had no identified high-risk exposure by our definition (Figure 1). An additional two antibody reactive tests were found to be false positives following laboratory EIA. Two further individuals were reactive to p24 antigen only and laboratory testing following the algorithm described above did not confirm HIV infection in either case. The overall HIV-prevalence in the study population was 1.8% (17/953) and was 3.1% (16/517) in “high-risk” individuals. The overall positive predictive value of a reactive rapid test in comparison to the laboratory gold standard was 80.9%. Confirmed HIV-positive individuals had a median CD4 count at diagnosis of 315 cells/ul (interquartile range [IQR]: 220–377).

Sixteen of 953 point of care tests (1.7%) were invalid on first rapid test. Two of these were reactive on immediate retesting and both were confirmed positive on laboratory testing. Six were non-reactive on immediate retesting and no further sample was sent. The remaining eight were EIA negative on laboratory retesting. Of the seventeen confirmed HIV-positive participants, two (12%) met our clinical definition for presumed recent HIV infection. Both also had a low HIV avidity index [16] consistent with recent infection (indices of 40% and 74% respectively). However, both were p24 antigen negative on rapid testing.

## Discussion

We describe results from a joint community and clinic-based programme using fourth generation rapid HIV tests. Screening was targeted towards at-risk individuals in a low prevalence setting and was able to identify new cases of HIV antibody positivity in 3% of those who self-reported themselves to be at “high-risk”. Near- patient tests performed in clinical and non-clinical settings offered the opportunity to increase availability of testing for hard-to-reach groups and allowed for rapid results [4]. We took the opportunity presented by rapid testing to incorporate prevention messages [17] and facilitate earlier access to care for those diagnosed positive [18].

Early diagnosis of HIV is recognized as an important public health intervention, with recent studies showing that a strategy of high population uptake of HIV testing linked to immediate initiation of ART could dramatically reduce HIV transmission and mortality in high prevalence settings [19]. In sub-Saharan Africa, HIV testing strategies include a broad mix of programmes, including voluntary counselling and testing (VCT), provider initiated testing (PITC) within health facilities, door-to-door testing and supervised self-testing in communities [20], [21]. However, in low HIV prevalence countries, programmes have lagged somewhat behind with particular shortcomings in identifying recently infected individuals [11]. New recommendations from the British HIV Association [22] and the Centers for Disease Control [23] have attempted to address this by recommending increased availability to HIV testing in non-clinical facilities and using novel technologies. Fourth generation rapid test kits have been suggested as an important tool in these strategies to increased the public health impact of HIV testing programmes by allowing early diagnosis and intervention in acute infection, and by facilitating decentralization of testing services into communities [21]. However, we identified a number of current limitations to their implementation.

In our programme both p24 antigen positive reactions were false positives and none of the two individuals presumed to have acute HIV infection were detected by p24 antigen. A previous study reporting performance of fourth generation rapid HIV test kits in community-based sites working with men who have sex with men in San Francisco reported a high sensitivity of 96% and specificity of 100% of the p24 component of Determine 4th generation [24]. These findings were neither borne out in our study nor in recent studies in Malawi [10] and the UK [9]. In Malawi, which is a higher HIV prevalence setting than ours, 846 individuals were tested in parallel with Determine 1/2 and found to be either HIV negative or serodiscordant on initial screening, the p24 portion of the Combo rapid test was falsely negative in all 8 acute HIV infections identified and there were 14 false positive p24 antigen tests [10]. Similarly, in the UK study, 62% of laboratory confirmed acute HIV infections were detected by the p24 component of Determine [9].

As in these studies, we found little advantage to the inclusion of p24 antigen and identified a number of disadvantages to the use of fourth generation antigen/antibody rapid test kits in community-based settings. Firstly, false positive diagnoses of seroconversion are likely to be high, particularly in low prevalence settings and could create a significant problem with point of care use of this assay, generating considerable anxiety for clients and lead to increased unnecessary loads on laboratories. Secondly, 1.7% of the tests we performed were invalid according to manufacturer's instructions. In almost all cases, we observed that the buffer failed to travel as far as the control line within the read time window. Invalid tests were observed throughout the study period and not confined to the first few weeks after training. This may be a factor of the additional length of strip used to accommodate the p24 strip. Finally, even those individuals identified by us as having recent HIV infection were already antibody reactive and p24 non-reactive on the rapid test when assessed, indicating that the additional value of the p24 component may be restricted to the first few days after transmission of the virus – a phenomenon that is well recognised elsewhere [25].

The main limitation of this programmatic evaluation is that only participants who had reactive or invalid point of care tests had laboratory samples analyzed. This means that we were unable to calculate the sensitivity, specificity and negative predictive value of the 4^th^ generation POCT kit. Despite this, we feel that it is valuable to present our findings as new fourth generation rapid tests are developed and piloted under programmatic conditions and future studies will be required to undertaken these evaluations. Secondly; our case definition of presumed recent HIV infection was primarily clinical. It included assessment of recent high-risk behavior from review of clinical notes and review of previous negative laboratory HIV testing results. HIV antibody avidity assays only became part of routine clinical practice in UK in the course of 2010 and as a result only 7 out of 17 newly diagnosed patients had samples referred for this test. Both patients meeting our clinical definition were confirmed as recent infections by confirmatory avidity testing. It is therefore possible that the prevalence of recent “high-risk” behavior may have been underestimated, meaning that some participants with confirmed infection may have not have been classified as having recent infection. Further, although we were able to access previous negative HIV test results in clinical files and from laboratory records, participants may have recently tested negative elsewhere.

We have found that community-based fourth generation HIV testing in low HIV prevalence settings may have limited additional benefit over programmes using third generation antibody only kits. In order to reach a balance between providing reliable and early diagnosis of HIV in the community and targeting testing programmes to hard-to-reach groups, we suggest a combined approach of outreach testing using third generation test kits with back up fourth generation laboratory-based EIA for individuals suspected to have acute HIV infection.
